# Scanner agnostic large-scale evaluation of MS lesion delineation tool for clinical MRI

**DOI:** 10.3389/fnins.2023.1177540

**Published:** 2023-05-19

**Authors:** Amalie Monberg Hindsholm, Flemming Littrup Andersen, Stig Præstekjær Cramer, Helle Juhl Simonsen, Mathias Gæde Askløf, Melinda Magyari, Poul Nørgaard Madsen, Adam Espe Hansen, Finn Sellebjerg, Henrik Bo Wiberg Larsson, Annika Reynberg Langkilde, Jette Lautrup Frederiksen, Liselotte Højgaard, Claes Nøhr Ladefoged, Ulrich Lindberg

**Affiliations:** ^1^Department of Clinical Physiology and Nuclear Medicine, Copenhagen University Hospital–Rigshospitalet, Copenhagen, Denmark; ^2^Department of Neurology, Copenhagen University Hospital–Rigshospitalet, Copenhagen, Denmark; ^3^Center for IT and Medical Technology, Copenhagen University Hospital–Rigshospitalet, Copenhagen, Denmark; ^4^Department of Radiology, Copenhagen University Hospital–Rigshospitalet, Copenhagen, Denmark; ^5^Department of Clinical Medicine, University of Copenhagen, Copenhagen, Denmark

**Keywords:** multiple sclerosis, white matter lesions (WML), automatic segmentation algorithm, clinical applicability, clinical dataset, heterogeneous dataset, multi-scanner

## Abstract

**Introduction:**

Patients with MS are MRI scanned continuously throughout their disease course resulting in a large manual workload for radiologists which includes lesion detection and size estimation. Though many models for automatic lesion segmentation have been published, few are used broadly in clinic today, as there is a lack of testing on clinical datasets. By collecting a large, heterogeneous training dataset directly from our MS clinic we aim to present a model which is robust to different scanner protocols and artefacts and which only uses MRI modalities present in routine clinical examinations.

**Methods:**

We retrospectively included 746 patients from routine examinations at our MS clinic. The inclusion criteria included acquisition at one of seven different scanners and an MRI protocol including 2D or 3D T2-w FLAIR, T2-w and T1-w images. Reference lesion masks on the training (*n* = 571) and validation (*n* = 70) datasets were generated using a preliminary segmentation model and subsequent manual correction. The test dataset (*n* = 100) was manually delineated. Our segmentation model https://github.com/CAAI/AIMS/ was based on the popular nnU-Net, which has won several biomedical segmentation challenges. We tested our model against the published segmentation models HD-MS-Lesions, which is also based on nnU-Net, trained with a more homogenous patient cohort. We furthermore tested model robustness to data from unseen scanners by performing a leave-one-scanner-out experiment.

**Results:**

We found that our model was able to segment MS white matter lesions with a performance comparable to literature: DSC = 0.68, precision = 0.90, recall = 0.70, f1 = 0.78. Furthermore, the model outperformed HD-MS-Lesions in all metrics except precision = 0.96. In the leave-one-scanner-out experiment there was no significant change in performance (*p* < 0.05) between any of the models which were only trained on part of the dataset and the full segmentation model.

**Conclusion:**

In conclusion we have seen, that by including a large, heterogeneous dataset emulating clinical reality, we have trained a segmentation model which maintains a high segmentation performance while being robust to data from unseen scanners. This broadens the applicability of the model in clinic and paves the way for clinical implementation.

## 1. Introduction

Multiple sclerosis (MS) is a chronic autoimmune disease of the central nervous system (CNS), which is the most common cause of long-term non-traumatic disability in young adults (Reich et al., 2018). The disease is characterized by inflammatory axonal demyelination and loss which manifests as focal lesions in the grey- and white matter of the CNS (Reich et al., 2018). Lesion activity is a primary biomarker for disease diagnosis, disease activity and treatment response (Smith et al., 2017; Thompson et al., 2018; Klistorner et al., 2021; Oship et al., 2022), and is monitored throughout the patient’s disease course by acquiring magnetic resonance images (MRI) of the brain and spinal cord (Sastre-Garriga et al., 2020). Manual assessment of MRI and registration of newly appearing or enlarging white matter lesions is a difficult and time-consuming task, which is routinely performed in the evaluation of the MR images. Despite volumetric lesion quantification being an established biomarker for disease progression, manual lesion segmentation is not carried out in clinical routine practice as it is an even more time-consuming task, prone to inter- and intra-rater variability (Altay et al., 2013). Recently, MS lesion segmentation has been of increasing interest in new research related to prediction of the clinical disease course (Zhao et al., 2017; Pruenza et al., 2019; Tousignant et al., 2019; Yoo et al., 2019; Pinto et al., 2020), risk of long-term disability (Popescu et al., 2013), treatment response (Wattjes et al., 2015), as well as establishing the MS diagnosis (Shoeibi et al., 2021).

Automatic MS lesion segmentation using artificial intelligence (AI) has been heavily researched in the last decade, and while segmentation performance has long been trailing manual segmentation (Commowick et al., 2018), recent models have met, and in few cases exceeded, the performance of clinical experts under controlled evaluation conditions (Carass et al., 2017a). This is in large part due to recent enhancements in both computer software and hardware, which has made it possible to train and apply deep learning models for biomedical segmentation tasks, with especially convolutional neural network (CNN) methods dominating the field of MS lesion segmentation (García-Lorenzo et al., 2009; Danelakis et al., 2018; Kaur et al., 2020; Zeng et al., 2020; Shoeibi et al., 2021; Zhang and Oguz, 2021). A popular CNN architecture is the U-net, originally presented for general biomedical image segmentation (Ronneberger et al., 2015), which is the architecture behind many leading models in international MS lesion segmentation challenges such as the MSSEG2016 Challenge and the ISBI2015 Longitudinal MS Lesion segmentation Challenge (Commowick et al., 2016; Carass et al., 2017a). Several promising DL models are published each year, obtaining incremental gains compared to the classical U-net. The main difference between the latest proposed methods is their focus on either quantifying both lesions and other brain structures in the same model (Cerri et al., 2021; McKinley et al., 2021; Weiss et al., 2021) or on enhancing performance on localized lesions, e.g., infratentorial and cortical, which often appear small and less hyperintense than other lesions. Regarding the latter La Rosa et al. (2020) proposed a U-net model for MS lesion segmentation, in which they had special focus on cortical lesions, assigning them with a separate label, while Rakiæ et al. (2021) has proposed a novel combination of a clinically implemented machine-learning model for brain structure measurement, with an attention-gated U-net to target cortical and infratentorial lesions. By fusing the outputs of the two networks, they found that they could improve segmentation of infratentorial and juxtacortical lesions by 14 and 31%, respectively.

A newly emerging branch of neural networks is transformer-based models, which has shown promising results in both language processing (Li G. Y. et al., 2023) and biomedical image segmentation (Hatamizadeh et al., 2022), and has quickly become state-of-the-art in some segmentation tasks such as head-and-neck cancer (Li G. Y. et al., 2023). So far, no implementations of transformer-based networks are readily available for MS lesion segmentation, and no large-scale clinical studies have been published. The nnU-Net is one of few ready-to-use segmentation frameworks available online. The model is distinguished by being a robust end-to-end framework, which includes pre-processing, post-processing and hyper-parameter optimisation and have obtained a high ranking in several biomedical segmentation challenges (Isensee et al., 2018).

Despite extensive research in automatic MS lesion segmentation, no model is broadly applied in clinical practice today, which is largely due to a lack of clinical testing and inadequate performance of the applied model. Model performance on clinical data is a challenge due to data heterogeneity compared to controlled and curated datasets, which is often used to develop and train AI-models. Clinical MRI often embrace a large variation of scanners, field strengths, image resolutions, protocols and intensity distributions, as well as patients with multiple stages and variations of the disease (Hindsholm et al., 2021). Furthermore, the performance of CNN models are generally challenged when applied to external datasets, and in many cases require re-training on a subset of local data (Valverde et al., 2019; Kamraoui et al., 2022). This is unsustainable in a clinical setting, as there is not necessarily capacity and knowledge, or high-quality delineated data, to train new models each time a new scanner is introduced. Studies show, however, that large and heterogeneous training datasets can increase model robustness when applied to external datasets (Mårtensson et al., 2020).

The aim of this study was to train and test a state-of-the-art MS lesion segmentation model on a large-scale heterogeneous dataset from clinical practice and evaluate segmentation performance in relation to clinical implementation. The latter will be achieved by testing model robustness to new scanners as well as an ablation study examining the number of necessary input modalities.

## 2. Materials and methods

### 2.1. Dataset

#### 2.1.1. Patients

We retrospectively included 2,817 consecutive patients (10,747 examinations) who had been referred for a routine MS-MRI examination from Copenhagen University Hospital–Rigshospitalet from January 2015 to October 2020. Patients were identified by linking The Danish Multiple Sclerosis Registry (Magyari et al., 2021) to their available clinical MRIs. All patients were adults (>18 years) and diagnosed with relapsing remitting MS following the time respective McDonald criteria (Thompson et al., 2018). From this dataset we selected a total of 746 examinations of 746 unique patients, using the following exclusion criteria: (1) the patients had to be scanned at one of the seven most prevalent scanner types in the dataset, (2) patient examinations were required to include a T2-weighted (T2-w) FLAIR sequence, a T2-w sequence, and a T1-w sequence without contrast, (3) protocols should be standardised within each scanner cohort, but not across cohorts, (4) only one examination per patient was included in the study chosen randomly from among the available time points. By selecting seven different scanner types, data acquired at both 1.5T and 3T scanners as well as 2D and 3D FLAIR images from a total of three different vendors were included. Additional protocol- and scanner specifications can be found in Table 1.

**TABLE 1:** Data acquisition parameters.

Manufacturer	Scanner	Field strength	No. of patients (Train/Validation/ Test)	Sequences				
				**FLAIR**		**T2w**	**T1w**	
				**2D**	**3D**	**2D**	**2D**	**3D**
Siemens	Avanto	1.5 T	115 (90/10/15)		x	x		x
Siemens	Verio	3 T	115 (90/10/15)		x	x		x
Siemens	Prisma fit	3 T	115 (90/10/15)		x	x		x
Siemens	Trio	3 T	109 (86/10/15)	x		x		x
GE medical systems	Optima MR450	1.5 T	113 (88/10/15)	x		x	x	
GE medical systems	Signa HDxt	1.5 T	56 (31/10/15)	x*	x*	x	x	
Philips medical systems	Achieva dStream	3 T	115 (90/10/15)	x		x	x	

*29% of the GE Signa HDxt patients were acquired with 2D FLAIR and 71% with 3D FLAIR.

The dataset was divided into three subsets each with an even proportion of patients from each of the seven scanners: training-dataset (*n* = 571 patients), validation-dataset (*n* = 70) and hold-out test-dataset (*n* = 100).

#### 2.1.2. Manual lesion masks

White matter lesion (WML) delineation of the training- and validation datasets was conducted by two of the authors (AH, UL) under supervision of two trained specialists with a total of >44 years of experience in delineating MS lesions (HJS, SPC) in a semi-automated, iterative process: A preliminary segmentation model provided segmentations which were corrected by AH and UL. As masks were corrected, the preliminary model was retrained to increase performance and decrease the need for correction. After this initial delineation, all masks were approved or corrected by two trained specialists (HJS, SPC). Lesion delineation was conducted slice-by-slice in original FLAIR image resolution using FSLeyes version 6.0.2 (McCarthy, 2023) and ITK-snap version 3.2 (Yushkevich et al., 2006) depending on the preference of the delineator.

Lesion delineation of the hold-out test-dataset was conducted manually in FSLeyes by contouring each lesion slice-by-slice in original FLAIR image resolution. Delineation was performed by a trained specialist (HJS), followed by a consensus-reading in ITK-snap by an experienced neuroradiologist with 20 years of experience (AL). Lesions in all three datasets were delineated on FLAIR-images while consulting T2-w images when needed.

#### 2.1.3. Ethics declaration

All patient-specific data was handled in compliance with the Danish data protection agency act no. 502. Collection of the retrospective dataset was approved by the National Committee on Health Research Ethics (protocol number 2117506). All patient-specific data were pseudo-anonymised and the GDPR (the European General Data Protection Regulation) was thereby fulfilled.

#### 2.1.4. Data pre-processing

The MR images were pre-processed in three steps inspired by Brugnara et al. (2020): (1) All inputs were reoriented to standard orientation [fslreorient2std, FSL (Jenkinson and Smith, 2001; Jenkinson et al., 2002)], (2) Brain-extraction was performed on all MRI-sequences [HD-BET (Isensee et al., 2019)], (3) T1-w and T2-w images were linearly (affine transformation with 12 degrees of freedom) registered to FLAIR-space using FSL FLIRT version 5.0 (Jenkinson and Smith, 2001; Jenkinson et al., 2002).

### 2.2. Segmentation network

We used the nnU-Net framework for lesion segmentation, which is an open-source toolkit comprised of pre-processing, training, inference, and post-processing (Isensee et al., 2018). The pre-processing steps included image normalisation according to the nnU-Net standard and resampling of all input-images to a common voxel-spacing. Network training was performed with T1-w, T2-w and FLAIR as input using 5-fold cross validation in the 3D full-resolution configuration of nnU-Net (for further description of network hyperparameters see Appendix A1) (Isensee et al., 2018). The final network was evaluated using the hold-out validation- (see below) and test-datasets. The model is available at https://github.com/CAAI/AIMS/.

### 2.3. Evaluation

#### 2.3.1. Comparison to state-of-the-art model

For comparison to state-of-the-art, publicly available segmentation models, we evaluated HD-MS-Lesions and the widely used LST-LGA.

HD-MS-Lesions is, similarly to our model, based on nnU-Net (Brugnara et al., 2020). HD-MS-Lesions deviates from the standard 3D nnU-Net in only two ways: Brugnara et al. (2020) utilise a soft dice loss as loss function and large patches of 128 × 128 × 128 voxels. The model was trained on a dataset of 334 patients with a standardised protocol from 3 scanners. Model input was 2D FLAIR, T1-w and T2-w images. 3D FLAIR images were not included in the HD-MS-Lesions training dataset.

The LST-lga is a lesion-growth algorithm which is part of the LST toolbox for SPM (we used version 3.0.0)^1^ (Schmidt et al., 2012). The algorithm first segments the T1 images into the three main tissue classes (CSF, grey matter and white matter). This information is then combined with the coregistered FLAIR intensities in order to calculate lesion belief maps. These maps are thresholded with a pre-chosen initial threshold (κ) into an initial binary lesion map which is subsequently grown along voxels that appear hyperintense in the FLAIR image. κ was set to its recommended default value of 0.3, as seen in previous studies (Cerri et al., 2021).

#### 2.3.2. Performance metrics

Lesion segmentation accuracy was assessed using the dice similarity coefficient (DSC) calculated at voxel-level. Lesion detection was assessed using the positive predictive value, also called Precision, the true positive rate called Recall and the lesion detection accuracy or F1-score calculated at lesion-level (Commowick et al., 2016). Further descriptions of all performance metrics can be found in Appendix A2.

To evaluate segmentation performance in relation to clinical implementation, the following two steps were carried out; testing of model robustness to new data as well as an ablation study examining model dependency on T1-w MRI and the effect of large datasets.

#### 2.3.3. Model robustness

Model robustness toward data from an unseen scanner was assessed by a leave-one-scanner-out (LOSO) cross-validation, where we trained seven models in total, each blinded to training data from one of the seven scanners in turn. The performance was compared to the main model on the test-dataset where we in each LOSO iteration only predicted the masks from the held-out scanner. We compared the difference to the main model using a *t*-test for each scanner individually.

#### 2.3.4. Ablation studies

The importance of including T1-w images in the model was evaluated by training a model with only FLAIR and T2-w MRI as input and comparing model performance to the main model on the validation dataset. This test was done, as T1-w images are not always part of the clinical protocol.

We then tested the importance of dataset size by training our model on different subsets of the total training-dataset and testing on the complete test-dataset. We created five data subsets by randomly sampling 10, 20, 40, 60, and 80% from the main dataset, while keeping the scanner distributions. Each subset was sampled five individual times to eliminate data-dependency. We furthermore sampled 10% of the main dataset from a single scanner, Achieva dStream, to investigate the effect of scanner heterogeneity in the training dataset.

To compare model performance of our model trained on our clinical dataset, with smaller, publicly available training datasets, we trained a model on two publicly available datasets and tested its performance on our test-dataset. The two datasets were from the ISBI2015 Longitudinal Multiple Sclerosis Lesion Segmentation Challenge (*n* = 5 patients with 4–5 examinations each) (Carass et al., 2017b) and the MSSEG MICCAI 2016 Challenge Dataset (*n* = 15 patients) (Commowick et al., 2018), respectively.

### 2.4. Implementation in clinical practice

The segmentation model has been implemented into a fully automatic pipeline ready for clinical application. Automatic lesion segmentation is carried out when requested after MR image acquisition and clinically relevant parameters from the lesion mask are extracted. If the MRI is a follow-up examination, the previous MRIs which have been processed by the model are collected and used for longitudinal analysis. The model output is then delivered back to the clinical imaging platform of choice. The model output is comprised of the binary lesion mask, the mask superimposed on a FLAIR image in a PACS preview as well as a PDF-report of summary results including total lesion load, lesion location, delineation examples and longitudinal changes from last examination (lesion appearance, disappearance and 30% enlargement).

## 3. Results

Patient demographics are displayed in Table 2. The training dataset had a total lesion count of 27,815 (mean = 48.7 lesions/patient, median = 37), and the test dataset of 4,978 (mean = 49.8 lesions/patient, median = 31). When examining lesion count per patient, 83% of the patients had more than 20 lesions (Table 3) in all three data subsets. Generally, all three subsets had the same distributions of lesion-load.

**TABLE 2:** Patient demographics.

Gender (f/m)	71%/29%
Mean age (median) (IQR)	46 years (45) (38–54)
MS phenotype	RRMS
Mean EDSS score (median) (IQR)	2.0 (1.5–3.5)
Mean disease duration from onset (range)	10.9 years (0–57.4)
Mean lesion volume (range)	15.6 mL (0.002–288.9)
Mean lesion count (range)	49 (2–323)

The EDSS score is the estimated score at time of MRI acquisition. Disease duration is measured from first diagnosis. EDSS, expanded disability status scale; MS, multiple sclerosis; RRMS, recurrent remitting MS.

**TABLE 3:** Number of lesions per patient as percentage of the total cohort.

Number of lesions	Training	Validation	Test
1	0.0% (0)	0.0% (0)	0.0% (0)
2	0.2% (1)	0.0% (0)	1.0% (1)
3	0.9% (5)	1.4% (1)	0.0% (0)
4	0.5% (3)	1.4% (1)	2.0% (2)
5–10	0.0% (0)	0.0% (0)	0.0% (0)
11–20	15.4% (88)	14.5% (10)	22.0% (22)
>20	83.0% (474)	82.6% (57)	75.0% (75)

Exact number of patients in parenthesis.

**FIGURE 1:**
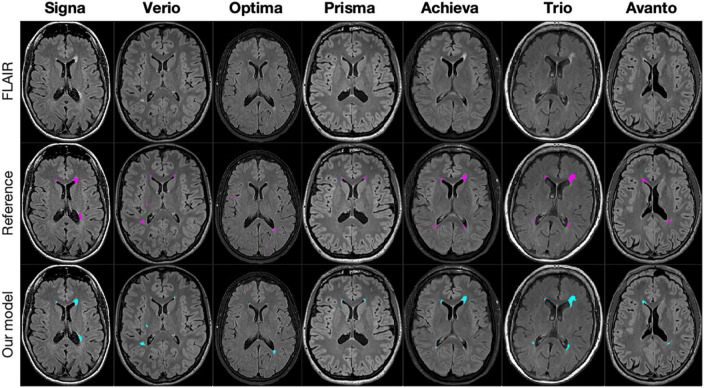
Slices from seven patients, one from each scanner, in the test dataset with delineations generated by our segmentation model (blue) and the reference delineation (pink).

We tested our segmentation model on the validation dataset (*n* = 70) and hold-out test dataset (*n* = 100) which were evenly distributed among the 7 scanner types. A visual illustration of a representative delineation result from each scanner is shown in Figure 1. Table 4 shows the average performance of our model and of the state-of-the-art model HD-MS-Lesions. The DSC and Recall scores were higher on the validation dataset than on the test dataset for both models. Our model outperformed HD-MS-Lesions in three out of four metrics on both the validation and test dataset with lower standard deviations. On the test dataset our model localised 70% of all lesions in the reference (recall), whereas HD-MS-Lesions localised 40%.

**TABLE 4:** Average metrics on the validation- and test dataset of 105 patients of our model and HD-MS-lesions as well as LST-LGA.

	DSC	Precision	Recall	*F1*
**Validation dataset**				
Our model	**0.82 (0.09)**	0.89 (0.08)	**0.83 (0.12)**	**0.86 (0.08)**
HD-MS-lesions	0.62 (0.20)	**0.93 (0.17)**	0.42 (0.21)	0.55 (0.21)
**Test dataset**				
Our model	**0.68 (0.11)**	0.90 (0.11)	**0.70 (0.15)**	**0.78 (0.11)**
HD-MS-lesions	0.57 (0.19)	**0.96 (0.13)**	0.40 (0.20)	0.54 (0.19)
LST-LGA	0.39 (0.21)	0.64 (0.25)	0.28 (0.17)	0.35 (0.18)

Each cell displays the mean (standard deviation) for each metric across all patients. DSC, dice similarity coefficient. Bold values mean the best performance.

**FIGURE 2:**
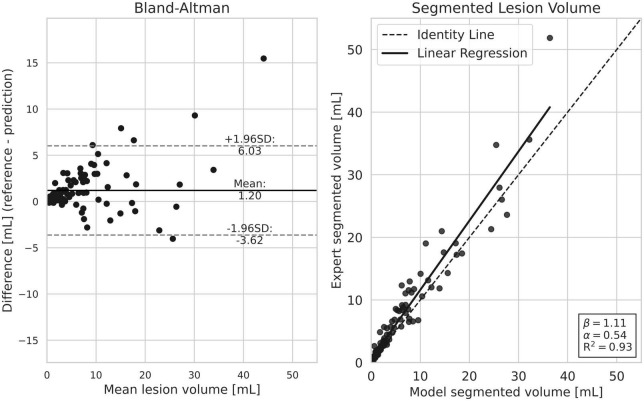
Comparison of lesion load estimation per patient between our model and the reference segmentation. Both plots show a slight tendency from our model to under-estimate total lesion volume. Generally, the discrepancy is smaller in subjects with a smaller lesion load.

Figure 2 displays the difference in estimated lesion volume per patient for the reference delineations and our model. We see a small tendency to underestimate lesion volume compared to the reference delineations, with an increasing discrepancy with total lesion load.

### 3.1. Scanner robustness

**FIGURE 3:**
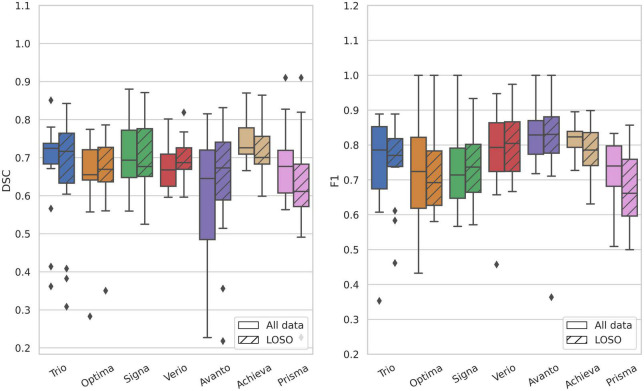
Evaluation metrics stratified by scanner type on the test dataset compared to the leave-one-scanner-out cross-validation experiment. Left, DSC; right, F1; striped, LOSO; no stripes, main model. In the leave-one-scanner-out LOSO-experiment each model was trained on data from 6 scanners and tested on the test data from the remaining scanner. There was no significant difference in performance between the full model and the LOSO-model for any of the scanners.

The average metrics per patient in the test dataset, grouped according to scanner, are displayed next to the results of the LOSO cross-validation experiment (striped) in Figure 3. Some variation is observed between the scanners, especially regarding the F1 scores in which the Optima, Prisma and Signa scanners obtain a slightly lower performance than the remaining cohorts. The results of the LOSO-experiment follow the performance of the full segmentation model with no significant differences (*P*-values > 0.05) for any of the scanners, indicating a high robustness to data from an unseen scanner. The corresponding results regarding Precision and Recall scores can be found in Appendix A4.

### 3.2. Ablation studies

Omitting the T1-w images in the model input resulted in nearly identical segmentation performance on the validation-dataset (DSC = 0.82, *F1* = 0.85) compared to the three-channel input. All segmentation results can be found in Appendix A3.

**FIGURE 4:**
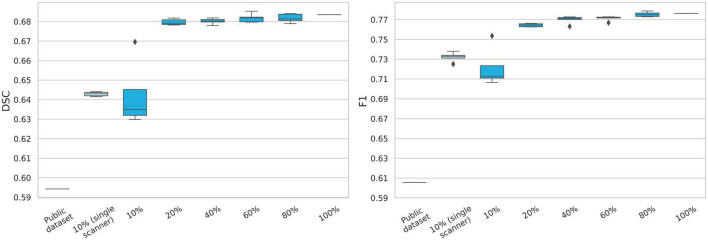
Evaluation of data size importance for model performance. The public data set consists of the two challenge datasets from ISBI2015 and MSSEG2016. The percentages refer to randomly sampled fractions of the main dataset, while keeping scanner distribution. A high increase in model performance is observed when 20% (*n* = 115) of the main training dataset is used, compared to 10% (*n* = 57). Model performance only slightly increases when more than 20% of the dataset used. The model clearly under-performs when trained on the two public datasets (*n* = 36). DSC, dice similarity coefficient.

The results of the dataset size ablation study can be found in Figure 4. A substantial difference in performance is observed between training on 10% or 20% and more of the main training dataset. From 20% (*n* = 115) and above, the segmentation performance is only incrementally improved. Training on 10% from a single scanner is seen to slightly outperform training on 10% from 7 different scanners. When training on the two combined public datasets (*n* = 36), both lesion segmentation performance (DSC) and lesion detection performance (F1) is lower than any fraction of our main dataset.

## 4. Discussion

In this study we collected a large MS MRI dataset reflecting the clinical reality from our Radiological Department with regards to scanner and protocol heterogeneity and used it to train and validate a state-of-the-art MS lesion segmentation model. We found that our model was able to find and segment MS lesions in the test dataset with a performance comparable to the literature (Aslani et al., 2019; La Rosa et al., 2020; McKinley et al., 2021), despite being a highly heterogeneous dataset including seven different scanners and protocols. The recorded agreement between our model-generated segmentation and the reference was furthermore comparable to many published accounts of inter-rater agreement, such as Cerri et al. (DSC = 0.59–0.69, 7 raters), Egger et al. (DSC = 0.66, 3 raters) and the 2015 ISBI segmentation challenge (DSC = 0.63, 2 raters) (Carass et al., 2017a; Egger et al., 2017; Cerri et al., 2021).

Many published datasets used for benchmarking are limited in size and protocol diversity, such as the MICCAI 2008 challenge dataset (*n* = 20/24 train/test) or the ISBI 2015 challenge dataset (*n* = 5/14 train/test) (van Ginneken et al., 2008; Carass et al., 2017a). Though the public datasets provide a great platform for model comparison, it does not emulate a models’ ability to segment MS lesions in a clinical setting, as it lacks both scanner, protocol and sequence heterogeneity. This point is underlined by training our model performance on just 10% of our dataset, and when training on two public datasets, where in both cases we saw a significant decrease in both DSC and F1-score. There was a large performance increase when training on 20% or more of our dataset, while keeping the scanner heterogeneity. Since performance did not increase significantly above 20%, this would indicate that a dataset of approximately *n* = 115 from heterogeneous scanners is sufficient for training a robust segmentation model.

As the goal of the present study was to evaluate segmentation performance in relation to clinical implementation, we chose to include both 2D and 3D FLAIR images acquired at both 1.5T and 3T MRI scanners, as used in our Radiological Department, and to not exclude images of low quality, unless our clinicians deemed the images unsuitable for an MS-assessment. Our dataset therefore includes motion artefacts and low signal intensities to mirror the clinical reality, which further complicates the segmentation task.

As a promising indication of the model performance in relation to clinical implementation, the model was robust to data from an unseen scanner in the LOSO-experiment. This is an important result, as MS clinics often include several different MRI scanners and patients are scanned at several clinics during their disease course. Gabr et al. (2020) performed a similar leave-one-centre-out experiment, in which they divided their 68 datasets into 17 partitions and also saw a low difference in DSC between centres. However, even though 68 centres participated in the study, they all used the same MRI protocol.

HD-MS-Lesions was trained on a relatively large dataset (*n* = 334) and reported very high segmentation metrics on their local test dataset (DSC = 0.88). However, their dataset was limited in data heterogeneity with a standardised protocol not including 3D FLAIR and T2-weighted images. We saw that the model generalised poorly to our test dataset, both the entire dataset (DSC = 0.57, *F1* = 0.53) and if tested only on the 2D FLAIR subset (DSC = 0.57, precision = 0.96, recall = 0.42, *F1* = 0.57). This underlines the need for heterogeneous training data if the model is to be applied to unseen data in clinic.

The fact that we achieve similar high performance metrics compared to previous studies, in spite of the inclusion of highly heterogeneous datasets, from 7 different scanner brands, including both 2D and 3D FLAIR sequences, and allowing for motion and other image artefacts, highlights the methodological improvements made toward clinical implementation of AI WML delineation in MS, and contests to a broader validity and applicability of our method.

### 4.1. Limitations

We experienced a difference in segmentation performance from our validation dataset to the test dataset, despite the two subsets being drawn from the same dataset. We attribute some of this variation to the difference in delineation practice between the train/validation dataset and the test dataset. We chose to decrease the extensive task of manual delineation by utilising a semi-automatic delineation approach on the train/validation dataset as seen in several publications (Le et al., 2019; Brugnara et al., 2020; Krüger et al., 2020). However, to avoid data spilling from the semi-automatic process, the test dataset was manually delineated by one rater and subsequently approved by another. We observed that the two approaches led to two different segmentation styles, similar to an inter-rater variability, as both approaches introduce a bias toward accepting the preliminary delineations during correction. Ideally, there should have been several manual delineations on the test dataset to account for the inter-rater variability, as ignoring it can result in a misleading impression of model performance. The segmentation model should therefore be validated clinically, which is our next step.

To optimise our model for clinical usability, we only included the most frequently used MRI sequences for MS examinations, with T1-w images without contrast being the only sequence which is not always included during examinations. However, as we saw a robustness to omitting T1-w images from the model input (DSC = 0.82 both with and without T1-w), a 2-channel version of the model could be used in these instances.

The patients in our dataset generally have a high lesion load (>20), which complicates the segmentation process. It would be relevant to investigate model performance on patients with a low lesion load, for use in cases of disease-onset. For subjects with a low total lesion volume, we saw that our model generally had a higher agreement with the reference models. One of the advantages of automatic lesion segmentation, besides enhanced segmentation speed, is that it is consistent and reproducible. This is especially important regarding longitudinal follow-up examinations, as it eliminates inter-rater bias between appointments.

Our dataset was limited in MS-subtype diversity, as all included patients had RRMS. In total, 85–90% of patients present with an RRMS phenotype from onset and regular MRI is part of the routine monitoring of disease modifying therapies, which guided the inclusion criteria.

### 4.2. Perspectives for future studies

The results of this study show, that when trained on a large, heterogeneous dataset, our AI-model can segment MS lesions from a clinical dataset, from a range of different scanners and protocols, with a performance comparable to more homogeneous models from literature. We furthermore show, that the model can maintain that high performance when applied to data from previously unseen scanners. This is promising for the implementation of the model in clinical practice, where the use of automatic segmentation could potentially reduce assessment time for the radiologist and eliminate some of the inter-rater variation which dominates MS-lesion segmentation. Before implementation, the model should be validated in clinical practice to ensure that delineation standards are satisfactory and that the output is a useful decision support for the radiologist.

## 5. Conclusion

In conclusion, we have demonstrated that an AI-model trained on a large heterogeneous clinical cohort can segment MS lesions with high performance results. Unlike previous studies, we included highly heterogeneous datasets which gives a more realistic portrayal of a real life clinical setting, broadening the general applicability of our method and paving the way toward a clinical implementation. We found that the lesion segmentation performance (DSC) and lesion detection performance (F1) was robust to data from unseen scanners, which is promising for clinical implementation. Future work will aim at determining the clinical value of the model in a clinical validation study.

## Data availability statement

The data analyzed in this study is subject to the following licenses/restrictions: The dataset contains patient identifiable information, and can therefore not be made publicly available. The dataset can be made available upon reasonable request. Requests to access these datasets should be directed to AH, amalie.monberg.hindsholm@regionh.dk.

## Ethics statement

The studies involving human participants were reviewed and approved by the National Committee on Health Research Ethics (approval number: 2117506). Written informed consent for participation was not required for this study in accordance with the national legislation and the institutional requirements.

## Author contributions

AH, FA, CL, UL, LH, JF, AEH, FS, and HL contributed to the design and conception of the study as well as overall direction and planning. AH, CL, MM, MA, and PM contributed to data acquisition, merging of databases, and dataset curation. SC, HS, and AL were in charge of reference delineations and had final approval, AH and UL assisted. AH, FA, UL, and CL contributed to the figures design and data analysis. AH wrote the manuscript draft with input from all authors. All authors contributed to the manuscript revision, read, and approved the submitted version.

## Footnotes

1
www.statisticalmodelling.de/lst.html
