# Supplementary material for: Scanner agnostic large-scale evaluation of MS lesion delineation tool for clinical MRI
Source: Front Neurosci. 2023 May 19;17:1177540. doi: 10.3389/fnins.2023.1177540 (PMC10235534; doi:10.3389/fnins.2023.1177540)

Supplementary Material

Scanner agnostic large-scale evaluation of MS lesion delineation tool for clinical MRI

**Amalie Hindsholm*^,Ϯ,1^, Flemming Littrup Andersen ^Ϯ,1^, Stig Præstekjær Cramer^1^, Helle Juhl Simonsen^1^,  Mathias Gæde Askløf^1^, Melinda Magyari^2^, Poul Nørgaard Madsen^3^, Adam Espe Hansen^4,5^, Finn Sellebjerg^2,5^, Henrik Bo Wiberg Larsson^1,5^, Annika Reynberg Langkilde^4^, Jette Lautrup Frederiksen^2,5^, Liselotte Højgaard^1,5^, Claes Nøhr Ladefoged ^Ϯ Ϯ,1^, Ulrich Lindberg ^Ϯ Ϯ,1^**

*** Correspondence:** Amalie Hindsholm: Amalie.monberg.hindsholm@regionh.dk

# Appendix A1: nnUNet

The nnU-Net is a deep learning network by Isensee et al (35), which is based on the popular U-net architecture for biomedical image segmentation (46). The nnU-Net is designed to require minimal optimisation to a new segmentation task, as it automatically extracts hyperparameters during network preprocessing. This is done by automatically defining a set of dataset fingerprints including image sizes, voxel spacing and class ratios. From this fingerprint, hyperparameters such as the appropriate patch-size, batch-size, number of feature maps and the convolutional kernel size are set.

After preprocessing our dataset had a common voxel spacing of [1.0, 0.6, 1.0] with a median matrix size of [134, 279, 140]. The patch size was set by the network to be [96, 224, 112] with a batch size of 2. The network was trained for 1000 epochs, using stochastic gradient descent as the optimiser and a learning rate of 0.01.

All computations were performed on a Linux machine running Ubuntu 20.04. The network was trained, using NVIDIA Titan Xp graphics cards from NVIDIA Corporation with CUDA Toolkit (version 11.6, https://developer.nvidia.com/cuda-toolkit). The network was implemented in Pytorch (version 1.11, www.pytorch.org) using Python (version 3.9, www.python.org. 3).

# Appendix A2: Evaluation metrics

The evaluation metrics were inspired by the metrics used in the MSSEG 2016 MS lesion segmentation challenge by MICCAI (23) including the Dice similarity coefficient (DSC), to measure lesion segmentation overlap between manual reference and prediction, and the F1-score to measure lesion detection. Furthermore, we included precision and recall for transparency, as they together make up the F1-score.

*DSC:*

The DSC is a voxel-wise metric, which measures the overlap of voxels in the prediction and reference:

$$DSC= \frac{2(G\cap A)}{|G|+|A|}$$

Where G denotes number of lesion voxels in the reference and A in the prediction.

*Precision:*

Also called the positive predictive value, is measured as the fraction of correctly detected lesions out of all algorithm-detected lesions:

$$precision= \frac{TP}{TP+FP}$$

Where TP (true positive) is the number of true positive lesions detected, and FP (false positive) is the number of falsely detected lesions by the algorithm as compared to the reference.

*Recall:*

Also called the lesion sensitivity of the algorithm or the true positive rate. It is measured as the fraction of true-positive algorithm-detected lesions of all lesions in the reference:

$$precision= \frac{TP}{TP+FN}$$

Where TP is the number of true positive lesions detected by the algorithm and FN (false negative) is the number of lesions in the reference, which were not detected by the algorithm.

*F1:*

The F1-score is a measure of lesion detection accuracy, and is calculated as the harmonic mean of the precision and recall:

$$F1= \frac{2 precision recall}{precision+recall}$$

# **Appendix** A3: Ablation Study

Metrics of the ablation study. All metrics are calculated as the mean across all validation patients. The standard deviation is noted in brackets.

|  | **DSC** | **Precision** | **Recall** | **F1** |
| --- | --- | --- | --- | --- |
| **2D nnU-Net** | 0.81 (0.10) | 0.83 (0.12) | 0.85 (0.12) | 0.83 (0.09) |
| **Our model without T1-w MRI** | 0.82 (0.09) | 0.90 (0.08) | 0.82 (0.13) | 0.85 (0.09) |

# **Appendix** A4: LOSO cross validation experiment

Box plots of the metric values Recall and Precision on the test dataset, of both the full-data model and the LOSO-model. The results of the LOSO-experiment follow the performance of the full segmentation model with no significant differences (P-values > 0.05) except for three instances: the recall values of the Achieva (P=0.019) and Prisma (P = 0.004) scanner models and precision of the Prisma model (P = 0.010). This indicates that especially the LOSO Prisma-model segments fewer true positive lesions than the general model (lower recall), but also fewer false positives (higher precision).


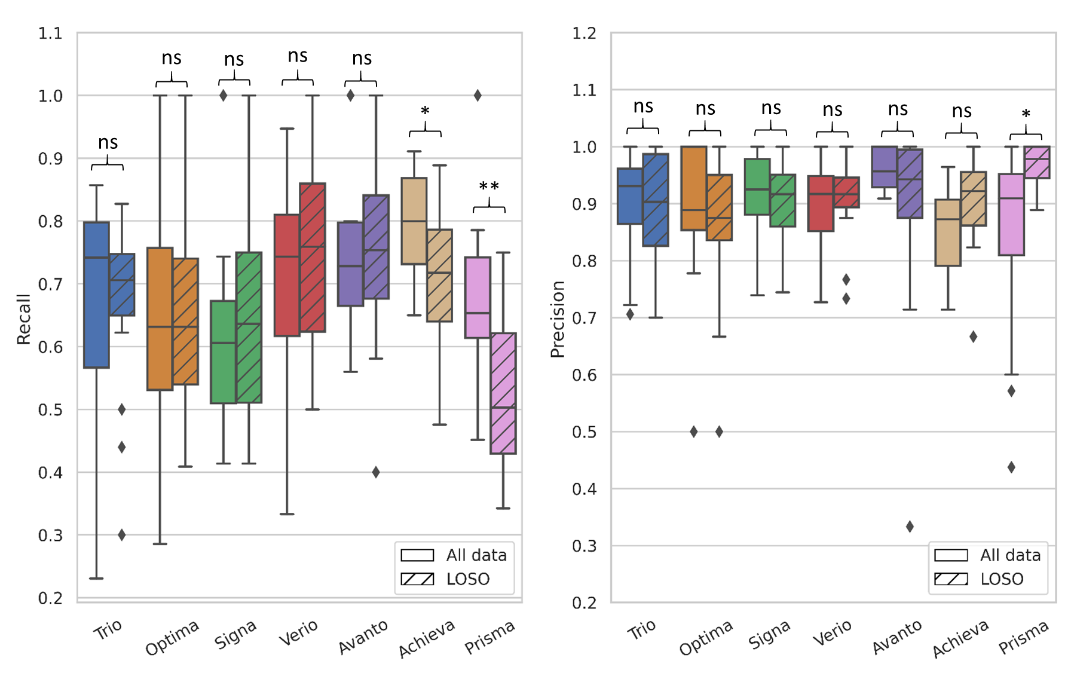

Supplement: Supplementary file 1 [file Data_Sheet_1.docx]
